# Association of neutrophil-lymphocyte ratio, platelet-lymphocyte ratio, and De Ritis ratio with mortality in renal cell carcinoma: A multicenter analysis

**DOI:** 10.3389/fonc.2022.995991

**Published:** 2022-11-24

**Authors:** Cathrine Keiner, Margaret Meagher, Dattatraya Patil, Kazutaka Saito, Arman Walia, Franklin Liu, Raksha Dutt, Nathan Miller, Sohail Dhanji, Ava Saidian, Fang Wan, Yosuke Yasuda, Yasuhisa Fujii, Hajime Tanaka, Viraj Master, Ithaar Derweesh

**Affiliations:** ^1^ Department of Urology, UC San Diego School of Medicine, La Jolla, CA, United States; ^2^ Department of Urology, Emory University School of Medicine, Atlanta, GA, United States; ^3^ Department of Urology, Tokyo Medical and Dental University, Tokyo, Japan

**Keywords:** renal cell carcinoma, inflammatory markers, prognostic markers, De Ritis ratio, neutrophil lymphocyte ratio, survival, all-cause mortality

## Abstract

**Background:**

Several markers of inflammation have been associated with oncologic outcomes. Prognostic markers are not well-defined for renal cell carcinoma (RCC). We sought to investigate the association of preoperative neutrophil-lymphocyte ratio (NLR), platelet-lymphocyte ratio (PLR), and De Ritis ratio with mortality in RCC.

**Methods:**

Multi-center retrospective analysis of patients undergoing surgery for RCC. Primary outcome of interest was all-cause mortality (ACM). Secondary outcomes were non-cancer mortality (NCM) and cancer-specific mortality (CSM). Elevated NLR was defined as ≥2.27, elevated PLR as ≥165, and elevated De Ritis ratio as ≥ 2.72. Multivariable cox regression analysis (MVA) was conducted to elucidate risk factors for primary and secondary outcomes, and Kaplan-Meier analysis (KMA) was used to evaluate survival outcomes comparing elevated and non-elevated NLR, PLR, and De Ritis ratio.

**Results:**

2656 patients were analyzed (874 patients had elevated NLR; 480 patients had elevated PLR and 932 patients had elevated De Ritis). Elevated NLR was a significant predictor of ACM (HR 1.32, 95% CI: 1.07-1.64, p=0.003) and NCM (HR 1.79, 95% CI: 1.30-2.46, p<0.001) in MVA. Elevated De Ritis was a significant predictor of ACM (HR 2.04, 95% CI: 1.65-2.52), NCM (HR 1.84, 95% CI: 1.33-2.55, p<0.001), and CSM (HR 1.97, 95% CI:1.48-2.63, p<0.001). KMA revealed significant difference in 5-year overall survival (OS) (48% vs. 68%, p<0.001), non-cancer survival (NCS) (69% vs. 87%, p<0.001), and cancer-specific survival (CSS) (60% vs. 73%, p<0.001) for elevated versus non-elevated NLR. For PLR, there was a difference in 5-year OS (51% vs. 61%, p<0.001) and CSS (60% vs. 73%, p<0.001) with KMA.

**Conclusions:**

Elevated NLR was independently associated with worse ACM and NCM, while elevated De Ritis was predictive for CSM in addition to ACM and NCM. These differences may be useful in refining risk stratification with respect to cancer-related and non-cancer mortality in RCC patients and deserve further investigation.

## Introduction

Renal cell carcinoma (RCC) is the 6th and 7th most common cancer in men and women, respectively, with approximately 73,750 new cases and over 14,000 deaths in the United States in 2020 (1). These numbers continue to rise in industrialized countries, in part, driven by increased utilization of diagnostic imaging, as well as the increased prevalence of risk factors such as obesity, cigarette smoking, and uncontrolled blood pressure (2). RCC is a metabolically driven disease (3). Indeed many of the risk factors for metabolic syndrome and drivers for renal functional degeneration may also be risk factors for renal carcinogenesis and modulate outcomes (3, 4).

Emerging reports suggest that the inflammatory and metabolic markers neutrophil-lymphocyte ratio (NLR), platelet-lymphocyte ratio (PLR), and De Ritis ratio (aspartate aminotransaminase [AST]/Alanine aminotransaminase [ALT]) may be associated with survival outcomes in RCC (5–9). The mechanisms underlying the relationship between these inflammatory markers and RCC are not well-understood, but may involve neutrophils and platelet release of inflammatory cytokines and growth factors including the proangiogenic vascular angiogenic growth factor (VEGF), contributing to tumor invasion and metastasis reflected in NLR and PLR (10), and alterations in cellular metabolism and aerobic glycolysis associated with cell proliferation reflected in changes in AST and ALT activity (11, 12).

As the incidence of RCC cases continues to rise, there is a need for the identification of readily obtainable blood-based markers associated with cancer-specific and non-cancer-related outcomes that can be incorporated into risk stratification tools to help guide clinical decision-making. We sought to examine the predictive ability of NLR, PLR, and De Ritis ratio for all-cause, non-cancer, and cancer-specific mortality following surgery for RCC utilizing a multi-institutional database.

## Methods

### Patient population

We performed a retrospective multicenter analysis of patients utilizing the International Marker Consortium of Renal Cancer (INMARC). Institutional Board Review approval was granted at each participating institution. Our protocols have been previously described (13, 14). Patients presenting with cortical neoplasms suspicious for RCC underwent staging cross-sectional imaging of the chest abdomen and pelvis. Type of surgery [Radical Nephrectomy (RN) or Partial Nephrectomy (PN)] and approach were driven by individual surgeons in a shared decision-making environment. All operations were conducted by urologic oncologists and follow-up was conducted according to guidelines (15, 16). We included patients with tumors staged I-IV as defined by the American Joint Committee on Cancer (AJCC) criteria. We excluded patients who did not undergo surgical therapy and those with non-cortical malignancy.

### Data collection and variable definitions

Baseline clinical data abstracted included information on patient age, gender, race, body mass index (BMI), and history of hypertension, diabetes mellitus (DM), or coronary artery disease (CAD). Tumor characteristics obtained included clinical tumor size, stage, and histology (17). All preoperative labs were collected within two weeks of the procedure. Laboratory values abstracted included preoperative and follow-up complete metabolic panel and complete blood count (CMP); estimated glomerular filtration rate (eGFR) was calculated by CKD-EPI equation (18).

NLR and PLR were calculated from preoperative complete blood count values. De Ritis ratio was defined as the ratio of AST to ALT. Optimum thresholds for high versus low NLR, PLR, and De Ritis ratio were determined using the receiver operating characteristic (ROC) curve with overall survival (OS) as the primary endpoint (19). We then selected thresholds with the highest Youden index. High and low NLR was defined as an NLR ratio ≥ 2.27 and NLR < 2.27, respectively. High and low PLR was defined as PLR ≥ 165 and PLR <165. High and low De Ritis ratio was defined as AST/ALT ≥ 2.72 and < 2.72, respectively.

### Statistical analysis

Our primary outcome of interest was all-cause mortality (ACM). Our secondary outcomes of interest were non-cancer mortality (NCM) and cancer-specific mortality (CSM).

Our primary outcome of all-cause mortality was compared between high and low NLR, PLR, and De Ritis ratio with Kaplan-Meier analyses, and differences were tested using log-rank tests. Multivariable cox regression analysis was conducted to test the association between high and low NLR, PLR, and De Ritis ratio with OS. MVA models were adjusted for age (continuous), race (other vs. African American), sex (female vs. male), BMI (continuous), coronary artery disease (no vs. yes), diabetes mellitus (no vs. yes), Tumor Stage (with Stage 1 as reference), metastasis (no vs. yes), Last eGFR < 45 ml/min/1.73m^2^ (no vs. yes), surgery type (partial vs. radical nephrectomy), NLR (≥ 2.27 vs. < 227), PLR (≥ 165 vs. < 165), and De Ritis ratio (≥ 2.72 vs. < 2.72).

Our secondary outcomes of NCM and CSM were each compared between high and low NLR, PLR, and De Ritis ratio groups with Kaplan-Meier analyses, and differences were tested using log-rank tests. Multivariable cox regression analysis was conducted to test the association between each secondary outcome with high and low NLR, PLR, and De Ritis ratio. The multivariable model for non-cancer mortality was adjusted for age (continuous), race (other vs. African American), sex (female vs. male) , BMI (continuous), coronary artery disease (no vs. yes), diabetes (no vs. yes), Tumor Stage (with Stage 1 as reference), metastasis (no vs. yes), Last eGFR < 45 ml/min/1.73m^2^ (no vs. yes), surgery type (partial versus radical nephrectomy), NLR (≥ 2.27 vs. < 2.27), PLR (≥ 165 vs. < 165) and De Ritis ratio (≥ 2.72 vs. < 2.72). The multivariable model for the outcome of cancer-specific mortality was controlled for age (continuous), race (other vs. African American), sex (female vs. male), BMI (continuous), diabetes (no vs. yes), Tumor Stage (with Stage 1 as reference), metastasis (no vs. yes), Last eGFR < 45 ml/min/1.73m^2^ (no vs. yes), surgery type (partial versus radical nephrectomy), NLR (≥ 2.27 vs. < 2.27), PLR (≥ 165 vs. < 165), and De Ritis ratio (≥ 2.72 vs. < 2.72). Analyses were conducted using IBM SPSS 28.0.1.0 with p < 0.05 considered significant.

## Results

### Baseline characteristics

**Table 1** demonstrates baseline demographics and clinical tumor characteristics. A total of 2,656 patients were analyzed. Median age was 60 years, and median follow-up time was 30 months. Of these patients, 1716 (64.6%) were male and 940 (35.4%) were female. 507 (19.1%) individuals identified as African American. Mean BMI was 29.9 ± 6.7 Kg/m^2^. Among our cohort, 925 (34.8%) patients had a history of hypertension, 358 (13.5%) patients had diabetes mellitus, and 331 (12.5%) patients had a history of coronary artery disease. Mean clinical tumor size was 5.6 cm and 1555 (58.5%) patients underwent partial nephrectomy (PN). The cohort consisted of 1529 (57.6%) patients with Stage I, 201 (7.6%) patients with Stage II, 583 (22.0%) with Stage III, and 343 (12.9%) patients with Stage IV disease. A total of 2202 (82.9%) patients had clear cell histology and 1246 (49.7%) individuals had high-grade tumors (Grade 3,4). There were 466 (17.5%) patients who presented with distant metastasis.

**Table 1:** Patient demographics and operative characteristics.

Variable	All patients (n=2656)
Median Age, years (IQR)	60 (IQR 51-68)
Sex	
Female	940 (35.4%)
Male	1716 (64.6%)
Race	
African American	507 (19.1%)
Non-African American	2137 (80.5%)
Not specified	13 (0.5%)
Mean BMI (Kg/m^2^, ± SD)	29.9 ± 6.7
HTN	925 (34.8%)
DM	358 (13.5%)
CAD	331 (12.5%)
Mean clinical tumor size (cm, ± SD)	5.6 ± 3.7
Surgery type	
Partial	1555 (58.5%)
Radical	1101 (41.5%)
Tumor Histology	
Clear Cell	2202 (82.9%)
Non-Clear Cell	454 (17.1%)
Tumor AJCC Stage	
Stage I	1529 (57.6%)
Stage II	201 (7.6%)
Stage III	583 (22.0%)
Stage IV	343 (12.9%)
Metastasis (M1)	466 (17.5%)
Nuclear Grade	
Low Grade (Grade 1, 2)	1320 (49.7%)
High Grade (Grade 3, 4)	1246 (49.7%)
Unclassified	90 (3.4%)
Baseline eGFR < 60 mL/min/1.73m^2^	923 (34.8%)
*De novo* eGFR < 60 mL/min/1.73m^2^	578 (21.8%)
*De novo* eGFR < 45 mL/min/1.73m^2^	457 (17.2%)
*De novo* eGFR < 30 mL/min/1.73m^2^	196 (7.4%)
Median preoperative NLR	2.50 95% CI: IQR 1.78 - 3.63]
NLR ≥ 2.27	874 (32.9%)
Median preoperative PLR	125 [IQR 91.40-174.80]
PLR ≥ 165	480 (18.1%)
Median preoperative AST/ALT	2.65 95% CI: IQR 1.25 - 2.90]
AST/ALT ≥ 2.72	932 (35.1%)
Median length of follow-up (months)	30 [IQR 10.7-74.8]
All-cause deaths (events)	628 (23.6%)
Non-cancer deaths (events)	293 (11.0%)
Cancer-specific deaths (events)	335 (12.6%)

For the entire cohort, median preoperative NLR was 2.50, PLR was 125, and AST/ALT was 2.65. A total of 874 (32.9%) patients had preoperative NLR≥2.27, 480 (18.1%) had preoperative PLR≥165, and 932 (35.1%) had preoperative AST/ALT≥2.72. At the last follow-up, 628 (23.6%) patients developed mortality from all causes, while 293 patients (11.0%) developed NCM, and 335(12.6%) patients developed CSM.

### Multivariable analyses for mortality outcomes

**Table 2** demonstrates multivariable analyses for predictors of ACM (**Table 2A**), NCM (**Table 2B**), and CSM (**Table 2C**). Age (HR 1.03 95% CI: 1.02-1.03, p<0.001), increasing tumor stage (HR=1.66-2.24, p<0.001), metastasis (HR 2.00 95% CI: 1.58-2.53] p<0.001), last eGFR<45 ml/min/1.73m² (HR 1.35, 95% CI: 1.11-1.64, p=0.003), NLR≥2.27 (HR 1.32 95% CI: 1.07-1.64], p=0.011), and De Ritis ratio≥2.72 (HR 2.04, 95% CI:1.65-2.52, p<0.001) were independently associated with worsened ACM. Independent predictors of worsened NCM were increasing age (HR 1.04 95% CI: 1.03-1.06, p<0.001), lower BMI (HR 0.97, 95% CI:0.95-1.00, p=0.03), Stage 3 vs. Stage 1 tumors (HR 1.45, 95% CI: 1.03-2.03, p=0.033), last eGFR < 45 ml/min/1.73m² (HR 1.53, 95% CI:1.14-2.05, p=0.004), NLR≥2.27 (HR 1.79, 95% CI: 2.46 - 1.30, p<0.001), and De Ritis ratio≥2.72 (HR 1.84, 95% CI:1.33-2.55, p<0.001). Age (HR 1.02, 95% CI: 1.00-1.03, p<0.001), stage T2 vs. stage T1 (HR 3.04, 95% CI:1.52-6.09, p=0.002), stage T3 vs. stage T1 (HR 3.94, 95% CI: 2.31-6.72, p<0.001), stage T4 vs. stage T1(HR 9.21, 95% CI: 5.28-16.05, p<0.001), non-clear cell histology (HR 1.45, 95% CI: 1.02-2.04, p=0.038), metastasis (HR 4.29, 95% CI: 2.97-6.21, p<0.001), high nuclear grade (HR 2.23, 95% CI:1.52-3.28, p<0.001), surgery type of PN versus RN (HR 1.61, 95% CI: 1.14-2.33, p = 0.007), and De Ritis ratio≥2.72 (HR 1.97, 95% CI:1.48-2.63, p<0.001) were all independently associated with worsened CSM.

**Table 2A:** Multivariable analysis for all-cause mortality (ACM).

Variable	Hazard ratio (HR)	95% CI Low	95% CI High	p
Age (continuous)	1.03	1.02	1.03	<0.001
Sex (female vs. male)	1.10	0.89	1.35	0.38
Race (other vs. AA)	1.17	0.86	1.46	0.41
BMI (continuous)	0.99	0.98	1.01	0.25
CAD (no vs. yes)	1.25	0.10	1.56	0.055
DM (no vs. yes)	1.18	0.91	1.53	0.211
Surgery Type (RN vs. PN)	1.14	0.92	1.41	0.25
Tumor stage (reference = Stage 1)				<0.001
Stage 2 vs. Stage 1	1.66	1.12	2.48	0.013
Stage 3 vs. Stage 1	2.25	1.73	2.93	<0.001
Stage 4 vs. Stage 1	1.32	1.07	1.64	<0.001
Metastasis (no vs. yes)	2.00	1.58	2.53	<0.001
Last eGFR < 45 ml/min/1.73m² (no vs. yes)	1.35	1.11	1.64	0.003
NLR ≥ 2.27 (no vs. yes)	1.32	1.07	1.64	0.011
PLR ≥ 165 (no vs. yes)	1.07	0.84	1.38	0.569
AST/ALT ≥ 2.72 (no vs. yes)	2.04	1.65	2.52	<0.001

**Table 2B:** Multivariable analysis for non-cancer mortality (NCM).

Variable	Hazard ratio (HR)	95% CI Low	95% CI High	p
Age (continuous)	1.04	1.03	1.06	<0.001
Sex (female vs. male)	1.30	0.95	1.79	0.10
Race (other vs. AA)	1.25	0.85	1.85	0.26
BMI (continuous)	0.97	0.95	1.00	0.03
CAD (no vs. yes)	1.10	0.75	1.63	0.62
DM (no vs. yes)	1.09	0.75	1.60	0.64
Surgery Type (RN vs. PN)	1.30	0.97	1.75	0.08
Tumor stage (reference = stage 1)				0.11
Stage 2 vs. Stage 1	1.33	0.79	2.24	0.28
Stage 3 vs. Stage 1	1.45	1.03	2.03	0.033
Stage 4 vs. Stage 1	1.51	1.51	0.96	0.077
Last eGFR < 45 ml/min/1.73m² (no vs. yes)	1.53	1.14	2.05	0.004
NLR ≥ 2.27 (no vs. yes)	1.79	1.30	2.46	<0.001
PLR ≥ 165 (yes vs. no)	1.27	0.85	1.89	0.25
AST/ALT ≥ 2.72 (no vs. yes)	1.84	1.33	2.55	<0.001

**Table 2C:** Multivariable analysis for cancer-specific mortality.

Variable	Hazard ratio (HR)	95% CI Low	95% CI High	p
Age (continuous)	1.02	1.00	1.03	0.013
Sex (female vs. male)	0.97	0.74	1.29	0.85
Race (other vs. AA)	1.070	0.73	1.57	0.73
BMI (continuous)	1.01	0.99	1.03	0.32
CAD (no vs. yes)	1.14	0.85	1.53	0.38
DM (no vs. yes)	1.35	0.95	1.94	0.10
Tumor stage (reference = Stage 1)				p<0.001
Stage 2 vs. Stage 1	3.04	1.52	6.09	0.002
Stage 3 vs. Stage 1	3.94	2.31	6.72	<0.001
Stage 4 vs. Stage 1	9.21	5.28	16.05	<0.001
Metastasis (no vs. yes)	4.29	2.97	6.21	<0.001
Nuclear Grade (3/4 vs. 1/2)	2.23	1.52	3.28	<0.001
Surgery (RN vs. PN)	1.61	1.14	2.33	0.007
Clear cell histology (yes vs. no)	1.45	1.02	2.04	0.038
Last eGFR < 45 ml/min/1.73m² (no vs. yes)	1.25	0.96	1.63	0.097
NLR ≥ 2.27 (no vs. yes)	1.08	0.80	1.46	0.60
PLR ≥ 165 (no vs. yes)	1.26	0.90	1.75	0.18
AST/ALT ≥ 2.72 (no vs. yes)	1.97	1.48	2.63	<0.001

### Kaplan-meier survival analyses

**Figure 1:**
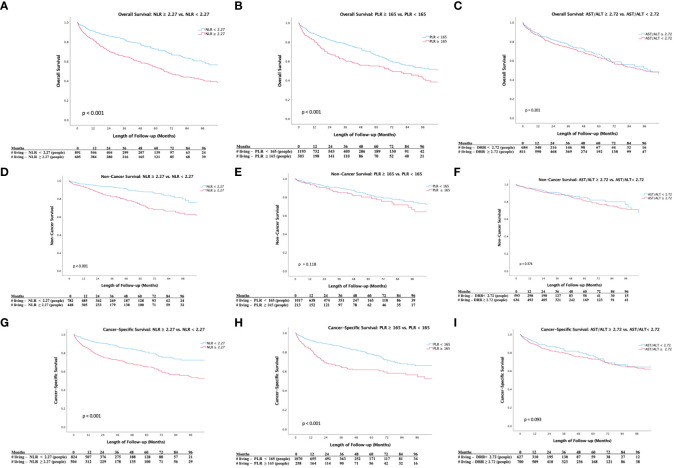
**(A)** Kaplan-Meier Survival Analysis of Overall Survival (NLR ≥ 2.27 vs. NLR <2.27). **(B)** Kaplan-Meier Survival Analysis of Overall Survival (PLR ≥ 165 vs. PLR< 165). **(C)** Kaplan-Meier Survival Analysis of Overal Survival (De Ritis Ratio ≥ 2.72 vs. De Ritis Ratio < 2.72). **(D)** Kaplan-Meier Survival Analysis of Non-Cancer Survival (NLR ≥ 2.27 vs. NLR <2.27). **(E)** Kaplan-Meier Survival Analysis of Non-Cancer Survival (PLR ≥ 165 vs. PLR< 165). **(F)** Kaplan-Meier Survival Analysis of Non-Cancer Survival (De Ritis Ratio ≥ 2.72 vs. De Ritis Ratio < 2.72). **(G)** Kaplan-Meier Survival Analysis of Cancer-Specific Survival (NLR ≥ 2.27 vs. NLR <2.27). **(H)** Kaplan-Meier Survival Analysis of Cancer-Specific Survival (PLR ≥ 165 vs. PLR< 165). **(I)** Kaplan-Meier Survival Analysis of Cancer-Specific Survival (De Ritis Ratio ≥ 2.72 vs. De Ritis Ratio < 2.72).

**Figures 1A–C** demonstrates Kaplan-Meier analyses for OS. We noted worsened 5-year OS in patients with NLR≥2.27 versus NLR<2.27 (48% vs 68%, p < 0.001; **Figure 1A**) and PLR≥165 versus PLR<165 (51% vs. 61%, p < 0.001; **Figure 1B**), while there was no significant difference in 5-year OS in patients with De Ritis ratio≥1.72 versus De Ritis ratio<1.72 (58% vs. 59%, p = 0.201; **Figure 1C**). **Figures 1D–F** demonstrates Kaplan-Meier analyses for NCS. We noted worse 5-year non-cancer survival (NCS) in patients with NLR≥2.27 versus NLR<2.27 (69% vs 87%, p<0.001; **Figure 1D**). Kaplan-Meier analyses showed no difference in NCS in patients with high versus low PLR (75% vs. 80%, p=0.118; **Figure 1E**) and De Ritis ratio (78% vs. 82%, p=0.374; **Figure 1F**). **Figures 1G–I** demonstrates Kaplan-Meier analyses for CSS. We noted worsened 5-year cancer-specific survival (CSS) among patients with NLR≥2.27 versus NLR<2.27 (60% vs. 73%, p<0.001; **Figure 1G**) and PLR≥165 versus PLR<165 (60% vs. 73%, p < 0.001; **Figure 1H**), while there was no significant difference in cancer-specific survival among patients with De Ritis ratio≥1.72 versus De Ritis ratio<1.72 (p=0.093; **Figure 1I**).

## Discussion

We investigated the utility of inflammatory markers as predictors of ACM, NCM, and CSM among patients with RCC who underwent partial or radical nephrectomy. We found elevated NLR to be an independent predictor for worsened ACM and NCM, while elevated De Ritis ratio was a significant predictor for all studied outcomes. PLR was not predictive of any of our outcomes. Our findings support further exploration of the utility of readily obtainable measures from common blood tests to predict outcomes at the time of presentation and guide decision-making.

We noted that NLR is significantly associated with ACM (HR=1.32, p=0.011) and NCM (HR=1.79, p<0.001), but not CSM (p=0.60). To our knowledge, our study is the first to investigate the association between NLR and NCM. The utility of NLR as a predictor of other outcomes in RCC has been investigated in a number of studies (5, 20–24). Our study confirms prior findings of the prognostic significance of NLR for OS, but there are conflicting results on the utility of NLR for predicting CSS. A single institution study by Bazzi et al. of 1970 non-metastatic patients similarly found elevated NLR to be a significant predictor of worse OS (p<0.0001) but not CSS (25). They did not report hazard ratios because NLR was modeled with nonlinear terms. Another study by Rajwa et al. of 455 patients with localized and non-localized disease similarly found elevated NLR was significantly associated with worse OS (HR=1.11, p=0.002) and not predictive of CSS (24). On the other hand, a study by Zapalla et al. of 495 patients and a meta-analysis by Shao et al. of 4133 patients found elevated NLR to be significantly associated with worse CSS (HR=2.62, p=0.043; pooled HR = 2.31, p< 0.001, respectively) (5, 23). We did not find NLR to be an independent predictor of CSS, however, heterogeneity between studies limits direct comparability. Zapala et al. excluded metastatic patients, whereas our cohort included patients with localized and non-localized disease (23). Additionally, the selection of a lower threshold to define high versus low NLR can influence results as more patients are proportionally assigned to the high NLR cohort. Our study defined a threshold of NLR greater than 2.27 while studies included in the meta-analysis used differing thresholds ranging from 2.17 to 3.5 (20–22, 26). Taken together, our findings and those of other published reports suggest that NLR may have utility as a predictor for overall survival or all-cause mortality. The divergent findings in regards to cancer-specific mortality and our finding that NLR was predictive of non-cancer mortality suggest that the association of NLR with survival outcomes may be driven by the effects of systemic disease as opposed to a reflection of direct oncological impact.

The current literature investigating the prognostic utility of De Ritis ratio for RCC has produced conflicting findings (7, 27), and in our study utilizing a threshold of 2.72, we found De Ritis ratio was significantly associated with all studied outcomes. A meta-analysis by Li et al. of 6,528 patients from 11 studies reported a significant association of high pretreatment De Ritis ratio with worse OS (HR 1.41, p<0.001) and CSS (HR 1.59, p<0.001) in patients with renal cell carcinoma (28). The meta-analysis, however, included three studies of patient cohorts treated non-surgically with tyrosine kinase inhibitors or targeted therapy. The largest study of surgically treated patients included in the meta-analysis analyzed a cohort of 2,965 patients with clear-cell localized disease and found that patients with a preoperative De Ritis ratio greater than three had significantly worse OS (HR 1.56, p=0.021) and CSS (HR.97, p=0.004). Differing from our results, a study by Canat et al. did not find an elevated De Ritis ratio above 1.5 to be an independent prognostic marker of OS (p=0.456) or CSS (p=0.293) in a cohort of 298 patients (27). They did, however, find an elevated De Ritis ratio to be associated with tumor characteristics such as renal vein invasion (p=0.025), renal capsule infiltration (p=0.015), and renal pelvis involvement (p=0.001) which have all been shown to be negative prognostic characteristics. Canat et al. used a threshold of 1.5 for a cohort of patients with only localized disease which limits direct comparability with our study. Additionally, our study with a large cohort of over 2,500 patients may have been better powered to detect a significant association. To our knowledge, the association of De Ritis ratio with non-cancer mortality has not yet been investigated. Our results support the utility of De Ritis ratio to predict overall survival, as well as cancer and non-cancer mortality for patients surgically treated for renal cell carcinoma.

We found elevated preoperative PLR was not significantly associated with any of the studied outcomes in our cohort of patients with non-metastatic and metastatic disease. Past studies that have reported an association between PLR and survival outcomes consist mostly of metastatic cohorts. The negative prognostic value of neutrophilia and thrombocytosis is well established for metastatic RCC with neutrophil and platelet counts being incorporated into validated prognostic tools such as the International Metastatic Renal Cell Carcinoma Database Consortium model, also known as the Heng Model (29). The Heng model, however, is validated for patients with metastatic RCC treated with targeted therapy. A study of patients with synchronous metastatic RCC by Yuk et al. reported an association between elevated PLR and poorer OS (HR=1.345; p<0.001) and CSS (HR=1.318, p<0.001) (30). Our study differs from the aforementioned work in that we included patients with localized disease and only patients who underwent extirpative surgery with partial or radical nephrectomy. One study that analyzed a cohort of patients with both localized and metastatic disease by Rajwa et al. found PLR to be a significant predictor of overall survival (HR=1.003, p=0.002), but not CSS in multivariable analyses (24). Differing from our study, they used a cutoff threshold of 168 to define elevated PLR. A meta-analysis by Wang et al. found elevated PLR was an effective prognostic marker of OS (pooled HR = 2.10, p = 0.001); however, only three of the seven studies included patients with localized disease (8). In their subanalysis of patients treated surgically, they did not find a significant association between OS and PLR (p=0.119). Taken together, while PLR may be an effective prognostic marker for advanced RCC, further investigation is needed to determine optimal thresholds and delineate the utility of PLR for more localized disease.

While our findings and their generalizability are strengthened by the diversity and size of our international cohort, there are limitations to our study. First, our study was a retrospective analysis which subjects our findings to biases inherent to such a design. Second, our study included only patients surgically treated for RCC, therefore, our study may not be generalizable to patients who choose non-surgical management options such as radiofrequency ablation, cryoablation, or active surveillance. Additionally, although we attempted to adjust for common variables, like comorbidity and tumor characteristics, there is the potential for other unmeasured factors that may have confounded our findings. Our data were collected from multiple international institutions and it is possible that differing treatment guidelines between institutions influence outcomes and are a potential confounder of our analysis that could not be controlled for.

## Conclusion

In our analysis, we noted that elevated NLR and De Ritis ratio were associated with worse overall survival and non-cancer mortality. Additionally, De Ritis ratio was significant prognostic marker of cancer-specific mortality. Elevated PLR was not predictive of any of the studied outcomes in our cohort. These differences may be useful in refining risk stratification with respect to cancer-related and non-cancer mortality in RCC patients and deserve further investigation.

## Data availability statement

The data analyzed in this study is subject to the following licenses/restrictions: We performed a retrospective multicenter analysis of patients utilizing the International Marker Consortium of Renal Cancer (INMARC) database. The data used to support the findings of this study have not been made available due to legal limitations of shared patient data across multiple international institutions. Requests to access these datasets should be directed to Ithaar Derweesh: iderweesh@gmail.com.

## Author contributions

ID and CK contributed to the conception and design of the study. DP, VM, and YF contributed to the acquisition of patients. CK, MM, RD, NM, DP, KS, AW, FL, SD, AS, and HT organized the database. CK and ID wrote the manuscript. All authors contributed to manuscript revision, read, and approved the submitted version.

## Funding

Stephen Weissman Kidney Cancer Research Fund.

## Conflict of interest

The authors declare that the research was conducted in the absence of any commercial or financial relationships that could be construed as a potential conflict of interest.

## Publisher’s note

All claims expressed in this article are solely those of the authors and do not necessarily represent those of their affiliated organizations, or those of the publisher, the editors and the reviewers. Any product that may be evaluated in this article, or claim that may be made by its manufacturer, is not guaranteed or endorsed by the publisher.
